# The simultaneous targeted Inhibition of ISG15 and HMGCR disrupts cancer stemness through metabolic collapse and induces synthetic lethality in pancreatic ductal adenocarcinoma

**DOI:** 10.1186/s13046-025-03561-x

**Published:** 2025-12-09

**Authors:** Jia Sun, Jia-Mei Wang, Qi Zhang, Wen-Ying Lin, Lin Tang, Shi-Yang Lu, Bai-Qiang Li, Zhen-Xian Du, Hua-Qin Wang

**Affiliations:** 1 Department of Biochemistry and Molecular Biology, China Medical University, Shenyang, 110122 China; 2 Department of Biochemistry and Molecular Biology, Nanjing University of Chinese Medicine, Nanjing, 210023 China; 3 Department of Laboratory Medicine, The First Hospital of China Medical University, Shenyang, 110001 China; 4 Department of Endocrinology & Metabolism, The First Hospital of China Medical University, Shenyang, 110001 China; 5 Department of University Hospital, Criminal Investigation Police, University of China, Shenyang, 110854 China; 6 Department of Endocrinology & Metabolism, The 1st affiliated Hospital, China Medical University, Shenyang, 110001 China

**Keywords:** ISG15, Statins, HMGCR, Synthetic lethal, Pancreatic ductal adenocarcinomas

## Abstract

Precision oncology, a transformative paradigm in cancer therapeutics, leverages molecular profiling to design personalized interventions. Building on our prior discovery of ISG15’s role in pancreatic cancer stem cell (CSC) generation, we identified co-upregulation of ISG15 and HMGCR in pancreatic ductal adenocarcinoma (PDAC). Dual suppression of ISG15 and HMGCR triggered synthetic lethality, effectively eradicating CSCs and impairing tumor progression. To exploit this synergy, we engineered a hyaluronic acid-coated liposomal nanoplatform (LNP-Kd/Statin) for co-delivery of ISG15-targeting siRNA and HMGCR-inhibiting statins. Mechanistically, ISG15 directly interacts with HMGCR, and the knockdown of ISG15 stabilizes the HMGCR protein by inhibiting K48 ubiquitin-proteasomal degradation, a process that occurs independently of ISGylation, paradoxically elevating cholesterol levels to sustain cell survival while depleting metabolic intermediates, thereby inducing vulnerability. Sterolomics profiling confirmed this metabolic rewiring, linking cholesterol flux collapse to CSC elimination. Our findings not only elucidate a novel ISG15-HMGCR regulatory axis in PDAC pathogenesis but also establish a nanotechnology-enabled strategy to disrupt cancer stemness through metabolic targeting. This work advances the translational potential of synthetic lethality in precision oncology for aggressive malignancies.

**Graphical Abstract:**

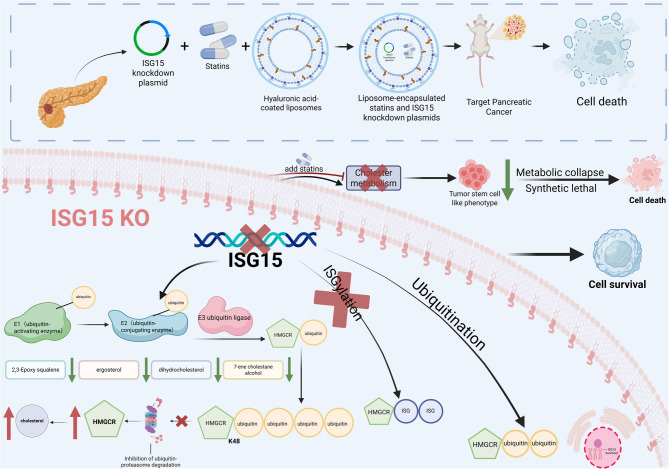

**Supplementary Information:**

The online version contains supplementary material available at 10.1186/s13046-025-03561-x.

## Introduction

Pancreatic cancer (PC) remains one of the most lethal malignancies, with a five-year survival rate below 10% due to late diagnosis, aggressive progression, and therapy resistance [1–3]. Cancer stem cells (CSCs), a subpopulation driving tumor initiation, metastasis, recurrence, and chemoresistance, are central to these challenges and represent a critical therapeutic target [4–6]. Synthetic lethality, a strategy where simultaneous inactivation of two genes induces cell death while individual inhibition does not, has emerged as a promising approach to eradicate CSCs while sparing normal cells [7, 8]. However, identifying actionable synthetic lethal pairs and their mechanisms in PC remains an unmet need.

Interferon-stimulated gene 15 (*ISG15*) is expressed at low levels in normal tissues but is significantly upregulated following viral infection or interferon (IFN) stimulation. Similar to ubiquitination, ISG15 can covalently modify substrate proteins through a process known as ISGylation, which is reversible through the action of specific de- ISGylating enzymes. Furthermore, ISG15 exists in both intracellularly and extracellularly in its monomeric form [9–12]. Although *ISG15* is overexpressed in PC and contributes to the maintenance of CSC phenotypes [13, 14], its role in regulating metabolic vulnerabilities remains poorly understood.

Metabolic vulnerability refers to the reprogramming of cellular metabolism, the inhibition of essential metabolic pathways, and the identification of targetable metabolic dependency that can be therapeutically exploited [15, 16]. It also involves utilizing tumor-specific metabolic preferences to facilitate the uptake of toxic analogues, thereby creating lethal metabolic traps [17, 18]. This approach represents a promising therapeutic intervention for cancer treatment. Intriguingly, our drug sensitivity screens revealed that *ISG15*-knockdown PC cells exhibit heightened sensitivity to statins, inhibitors of 3-hydroxy-3-methylglutaryl-coenzyme A reductase (*HMGCR*), the rate-limiting enzyme in cholesterol biosynthesis [19, 20]. While *ISG15* silencing or statin monotherapy partially suppressed CSC formation, their combination induced near-complete CSC eradication and cell death. Clinically, tissue microarray analyses of human and murine PC specimens confirmed that elevated ISG15 and HMGCR protein levels correlate with advanced tumor grades, despite unchanged mRNA expression post-*ISG15* knockdown. This paradox highlights post-translational regulation as a key mechanistic layer.

Nanotechnology has revolutionized drug delivery in PC, enabling targeted therapy and reduced systemic toxicity [21, 22]. Notably, nano-formulated irinotecan has entered clinical use [23–25]. In light of this, we engineered a hyaluronic acid (HA)-coated liposomal nanoplatform for co-delivering statins and *ISG15*-targeting plasmids. This system synergistically disrupts cholesterol metabolism and CSC stemness, achieving potent tumor suppression in organoids and xenograft models.

Mechanistically, *ISG15* knockdown stabilized HMGCR protein by blocking the K48 ubiquitin-proteasomal degradation, elevating cholesterol levels to sustain CSC survival. However, sterolomics revealed a concurrent depletion of cholesterol biosynthesis intermediates (e.g., desmosterol, squalene oxide), rendering cells metabolically vulnerable. Thus, dual targeting of ISG15 and HMGCR via our nanoplatform triggers a metabolic trap: while accumulated cholesterol transiently supports survival, the collapse of intermediate flux cripples CSC maintenance and tumor growth. These findings not only unravel a synthetic lethal ISG15-HMGCR axis in PC but also pioneer a nanotechnology-driven strategy to dismantle CSC-driven malignancy through metabolic reprogramming (Graphical Abstract).

## Materials and methods

### Materials

Glyceryl monostearate (GMS), soy phosphatidylcholine (SPC), cholesterol, DSPE-PEG-HA (hyaluronic acid-conjugated lipid), dimethyldioctadecyl ammonium bromide (DDAB), and 1,1’-dioctadecyl-3,3,3’,3’-tetramethylindocarbocyanine perchlorate (Dil) were procured from Shanghai Yuanye Bio-Technology (Shanghai, China). The *ISG15*-targeting RNAi plasmid (shRNA sequence: 5′- CATGTCGGTGTCAGAGCTGAA − 3′) was synthesized by Shanghai Genechem Co., Ltd. Atorvastatin calcium, Cycloheximide(CHX), Aloxistatin (E64d), MG132, Pepstatin A (purity > 98%) was purchased from Selleck.cn (Shanghai, China). Lentiviruses carrying ISG15-WT and ISG15 -MUT(G156/157A) were constructed by Shanghai Genechem Co.,Ltd.

### Preparation of LNP-Kd/Statin

Cationic liposomal nanoparticles (LNPs) encapsulating *ISG15*-RNAi plasmid and atorvastatin (LNP-Kd/Statin) were prepared using a thin-film hydration-sonication method. Briefly, a lipid mixture containing GMS (10 mg), SPC (20 mg), cholesterol (10 mg), DDAB (10 mg), DSPE-PEG-HA (1 mg), and atorvastatin (1 mg) was dissolved in 10 mL of chloroform/ethanol (5:1, v/v). The organic solvent was evaporated under reduced pressure (Rotavapor R-300, Büchi, Switzerland) to form a thin lipid film, which was hydrated with nuclease-free ddH_2_O (10 mL) under gentle agitation. The suspension was sonicated (40 kHz, 300 W) for 10 min at 45 °C and extruded through a 0.45-µm polycarbonate membrane to obtain blank LNP-Statin. For plasmid loading, 0.65 mg of *ISG15*-RNAi plasmid (in 10 mM Tris-EDTA buffer, pH 7.4) was added dropwise to the LNP-Statin solution under magnetic stirring (500 rpm, 30 min). Control nanoparticles (LNP and LNP-Kd) were prepared analogously.

### Fluorescent labeling

For cellular uptake studies, Dil (1 mg) was co-encapsulated with atorvastatin during lipid film formation to prepare Dil@LNP-Kd/Statin.

### Physicochemical characterization

Size and Zeta Potential: Hydrodynamic diameter, polydispersity index (PDI), and zeta potential were measured in triplicate using dynamic light scattering (DLS, Zetasizer Nano ZS90, Malvern Instruments, UK).

Morphology: Samples stained with 2% phosphotungstic acid were imaged by transmission electron microscopy (TEM, JEM-2100plus, JEOL, Japan) at 100 kV.

Drug Loading and Encapsulation Efficiency: After centrifugation (13,000 ×g, 40 min), free drug in the supernatant was quantified by UV-vis spectroscopy (290 nm). Encapsulation efficiency (EE) and drug loading (DL) were calculated as:

$$\begin{aligned}\mathrm{EE}\;(\%)&=({\mathrm{Drug}}_{\mathrm{feed}}-{\mathrm{Drug}}_{\mathrm{supernatant}})\\&/{\mathrm{Drug}}_{\mathrm{feed}}\times100\end{aligned}$$

$$\begin{aligned}\mathrm{DL}\;(\%)&=({\mathrm{Drug}}_{\mathrm{feed}}-{\mathrm{Drug}}_{\mathrm{supernatan}})\\&/\mathrm{Total}\;\mathrm{nanoparticle}\;\mathrm{weight}\times100\end{aligned}$$

Results:

$$\begin{aligned}&\mathrm{EE}\sim\mathrm{atorvastatin}\sim\;=\;58.07\;\pm\;6.71\%;\;\\&\mathrm{DL}\sim\mathrm{atorvastatin}\sim\;=\;1.12\;\pm\;0.10\%;\;\end{aligned}$$

$$\begin{aligned}&\mathrm{EE}\sim\mathrm{plasmid}\sim\;\approx\;100\%;\;\\&\mathrm{DL}\sim\mathrm{plasmid}\sim\;=\;1.25\%\end{aligned}$$

### Quantitative calibration

A linear correlation (*R*² = 0.998) between UV absorbance (290 nm) and atorvastatin concentration (7.81–500.00 µM/mL) was established (Figure S1).

### Cell culture and maintenance

Human pancreatic ductal adenocarcinoma (PDAC) cell lines BxPC3 (ATCC^®^ CRL-1687™) and SW1990 (GeneChem, Shanghai) were cultured in Dulbecco’s Modified Eagle Medium (DMEM; Gibco, Thermo Fisher Scientific) supplemented with 10% heat-inactivated fetal bovine serum (FBS; Sigma-Aldrich), 100 IU/mL penicillin, and 100 µg/mL streptomycin (Sigma-Aldrich). Cells were maintained in a humidified incubator (37 °C, 5% CO₂) with medium replenishment every 48–72 h. All cell lines underwent routine mycoplasma screening (MycoAlert™, Lonza) and were authenticated via short tandem repeat (STR) profiling prior to experimentation.

### Cytotoxicity assay

Cell viability was assessed using the CCK-8 assay. Briefly, BxPC3 and SW1990 cells were seeded into 96-well plates at a density of 4 × 10³ cells/well in 100 µL complete medium. After 12 h of attachment, cells were treated with escalating concentrations of LNP-Kd/Statin (0.1–100 µg/mL) or control nanoparticles. Following 48 h of incubation, 10 µL CCK-8 reagent (Dojindo Laboratories) was added to each well, and plates were incubated for 2 h at 37 °C under 5% CO₂. Absorbance was measured at 450 nm using a microplate reader (BioTek Synergy H1), with background subtraction at 650 nm. Cell viability was calculated as:

Viability (%)=(Atreatment − Ablank)/(Acontrol − Ablank)×100.

Data represent mean ± SD of three independent experiments (*n* = 6 replicates per group).

### In vitro cellular uptake analysis

The intracellular delivery efficiency of LNP-Kd/Statin was evaluated using confocal laser scanning microscopy (CLSM; Leica TCS SP8, Wetzlar, Germany). BxPC3 cells were seeded into 12-well plates containing glass coverslips at a density of 5 × 10⁴ cells/well and cultured until reaching 60% confluence. Cells were then incubated with Dil-labeled LNP-Kd/Statin (equivalent to 1 µg/mL Dil) in serum-free DMEM for 8 h at 37 °C under 5% CO₂. Following incubation, cells were washed thrice with ice-cold PBS, fixed with 4% paraformaldehyde (15 min), and counterstained with DAPI (1 µg/mL, 5 min) for nuclear visualization. Fluorescence signals were acquired using a 63× oil immersion objective with excitation/emission wavelengths set to 549/565 nm (Dil) and 358/461 nm (DAPI). Z-stack images (0.5 μm slices) were reconstructed using Leica LAS X software to assess subcellular nanoparticle distribution.

### Sterolomics profiling and quantitation

Sterolomic analysis was performed by LipidALL Technologies following established protocols [26]. Briefly, total lipids were extracted from pancreatic cancer tissues using a modified Bligh-Dyer method with the following adaptations: (1) tissue homogenization in chloroform/methanol/water (2:2:1.8, v/v/v) containing 0.01% butylated hydroxytoluene (BHT) as antioxidant; (2) phase separation facilitated by adding 1 M KCl to a final chloroform/methanol/water ratio of 1:1:0.9. The lipid extract was subjected to saponification in 500 µL of 1 N ethanolic potassium hydroxide (KOH) containing 5 µg butylated hydroxytoluene (BHT) at 37 °C for 1 h under constant shaking (1200 rpm). Subsequently, 50 µL of a deuterated internal standard mixture was spiked into each sample, comprising the following components:*d*₆-Lanosterol (10 ng), *d*₅-Zymosterol (10 ng), *d*₇-Lathosterol (10 ng), *d*₇−7-Dehydrocholesterol (10 ng), *d*₇-Sitosterol (10 ng) and *d*₆-Cholesterol (50 ng; Avanti Polar Lipids, Alabaster, AL, USA).

### LC-MS/MS analysis

Derivatized sterols were separated on a Shimadzu 40 × 3B UPLC system using a Kinetex C18 column (2.1 × 100 mm, 1.7 μm; Phenomenex) with mobile phases: (A) 0.1% formic acid in water and (B) 0.1% formic acid in acetonitrile/isopropanol (1:1). A gradient elution program was applied as 0–2 min: 60% B (isocratic), 2–15 min: Linear increase to 95% B, 15–18 min: Re-equilibration at 60% B. Detection was performed on a Sciex QTRAP 6500 + mass spectrometer in positive MRM mode. Quantitation was achieved via isotope dilution method using scheduled MRM (sMRM) with Analyst 1.7.1 software. Calibration curves (R² >0.99) were constructed for each sterol using analyte/internal standard peak area ratios [26].

### CRISPR/Cas9-mediated ISG15 knockout in pancreatic cancer cells

A dual single-guide RNA (sgRNA) system was employed to disrupt *ISG15* via CRISPR/Cas9 genome editing. The sgRNAs (sgRNA-1: 5′- GTGGTGGACAAATGCGACGAA-3′; sgRNA-2: 5′- GCACCGTGTTCATGAATCTGC-3′) were designed to flank exon 2 of *ISG15*, maximizing frameshift potential. A donor vector containing homology arms (1.5 kb each) was constructed to replace the target sequence with a puromycin resistance gene (*PuroR*) and enhanced green fluorescent protein (*EGFP*) cassette via homologous recombination (HR).

The dual sgRNA/Cas9 plasmid and donor vector were co-delivered into BxPC3 and SW1990 cells using lentiviral transduction (MOI = 10). Control cells received an empty sgRNA vector. At 72 h post-transduction, cells were selected with puromycin (2 µg/mL) for 7 days. *ISG15* knockout was validated through Genomic PCR (Amplification of the edited locus), qRT-PCR (*ISG15* mRNA levels were reduced by > 95% in knockout (KO) clones). Western Blot (Anti-ISG15 antibody confirmed protein ablation). EGFP-positive clones were isolated via fluorescence-activated cell sorting (FACSAria III, BD Biosciences) and expanded for functional assays.

### Colony formation assay

For the plate colony formation assay, 200 cells/well were seeded into six-well plates (Corning, Acton, MA, USA) and cultured for 2 weeks. The cells were then fixed with 4% paraformaldehyde for 15 min and stained with 0.1% crystal violet. The colony (containing more than 50 cells) numbers were determined with optical microscopy.

### Sphere formation assay

The cells in the logarithmic growth phase were harvested and resuspended in serum-free DMEM/F12 medium supplemented with 20 mg/ml EGF (Sigma-Aldrich, Saint Louis, MO, USA), 5 µg/ml Insulin (Sigma-Aldrich), and 2% B27(Invitrogen, Carlsbad, CA, USA). Cells were plated in six-well Corning Spheroid Microplates. The media were changed every 3 days, images of cells were taken under an inverted microscope and the numbers of spheroid were counted.

### Nude mouse xenograft experiments

1 × 10^7^ cells of BxPC3 cells were injected subcutaneously into BALB/c-nu/nu mice. 10 days after vaccination; Tumor-bearing mice were randomly divided into 4 groups: liposome/control, liposome/knockdown ISG15, liposome/statin drug, and liposome/knockdown ISG15 statin. Once daily for 2–3 weeks. During the experiment, the physiological conditions such as mental state, feeding, drinking, appearance, and body temperature of the mice were observed and recorded, and the dosing volume was adjusted according to the body weight, and the body weight and survival rate of the mice were recorded. All animal procedures were approved by and compiled with the guidelines of the Institutional Animal Care Committee of Nanjing University of Chinese Medicine(202403A057).

### Western blot analysis

The cells were washed twice with ice-cold PBS, scraped in ice-cold RIPA lysis buffer(Beyotime).Clarified lysate protein concentrations were determined using Bradford reagent (Bio-Rad) before sample normalization for SDS-PAGE electrophoresis using 4–12% Bis-Tris NuPage gels at 200 V for 60 min. Electrophoresed samples were transferred on nitrocellulose membranes and blocked using 3% BSA dissolved in TBST (50 mM Tris [pH 8.0, 4 °C], 150 mM NaCl, and 0.1% Tween-20) for 1 h at room temperature. Membranes were then incubated with primary antibody overnight at 4 °C.All antibody were provided by Cell Signaling Technology (CST). The next day, membranes were washed three times in TBST before the addition of secondary antibody conjugated with horseradish peroxidase (HRP) or streptavidin-HRP. Membranes were further washed three times with TBST before ECL exposure.

### RT-qPCR

Total RNA from cells were extracted using RNeasy^®^Mini Kit (QIAGEN) according to the instructions of manufacturer. After reverse transcription (Promega), standard RT-qPCR was performed to detect HMGCR mRNA in the precipitates. The primers are designed and synthesized by the Sangon Biotech.

### Flow cytometry

Cancer stem cell populations were analyzed by flow cytometry. BxPC3 and SW1990 cells in the logarithmic growth phase, including both control and ISG15 knockdown (KD), were treated with DMSO or statins for 24 h. After treatment, the cells were harvested and dissociated with 0.25% EDTA-free trypsin to generate single-cell suspensions. The cells were then washed twice with PBS (centrifugation at 300 × g for 5 min each) and adjusted to a concentration of 1 × 10 ^6^ cells per 100 µL in PBS containing 1% BSA. Fluorochrome-conjugated antibodies and corresponding isotype controls (CD133-APC and CD44-APC, Elabscience) were added according to the manufacturer’s instructions, and staining were performed in the dark at 4 °C for 30 min. After incubation, cells were washed once with PBS and resuspended in 400 µL PBS for analysis. Data acquisition was carried out on a BD FACSCanto II flow cytometer, and subsequent analysis was performed using FlowJo software (version 10.0).

### Statistics

Statistical parameters and significance are reported in the figures and the figure legends. The statistical significance of the difference was analyzed by ANOVA and post hoc Dunnett’s test. All experiments were repeated three times, and data were expressed as the mean ± SD (standard deviation). Significance is indicated by asterisks: *p < 0.05, **p < 0.01, ns. not significant. All charts are completed in Prism10 and the mechanism diagram was created in Biorender (Agreement number:PG28T9IOLMA).

## Results

### Loss of ISG15 sensitizes pancreatic cancer to statins via synthetic lethality

**Fig. 1:**
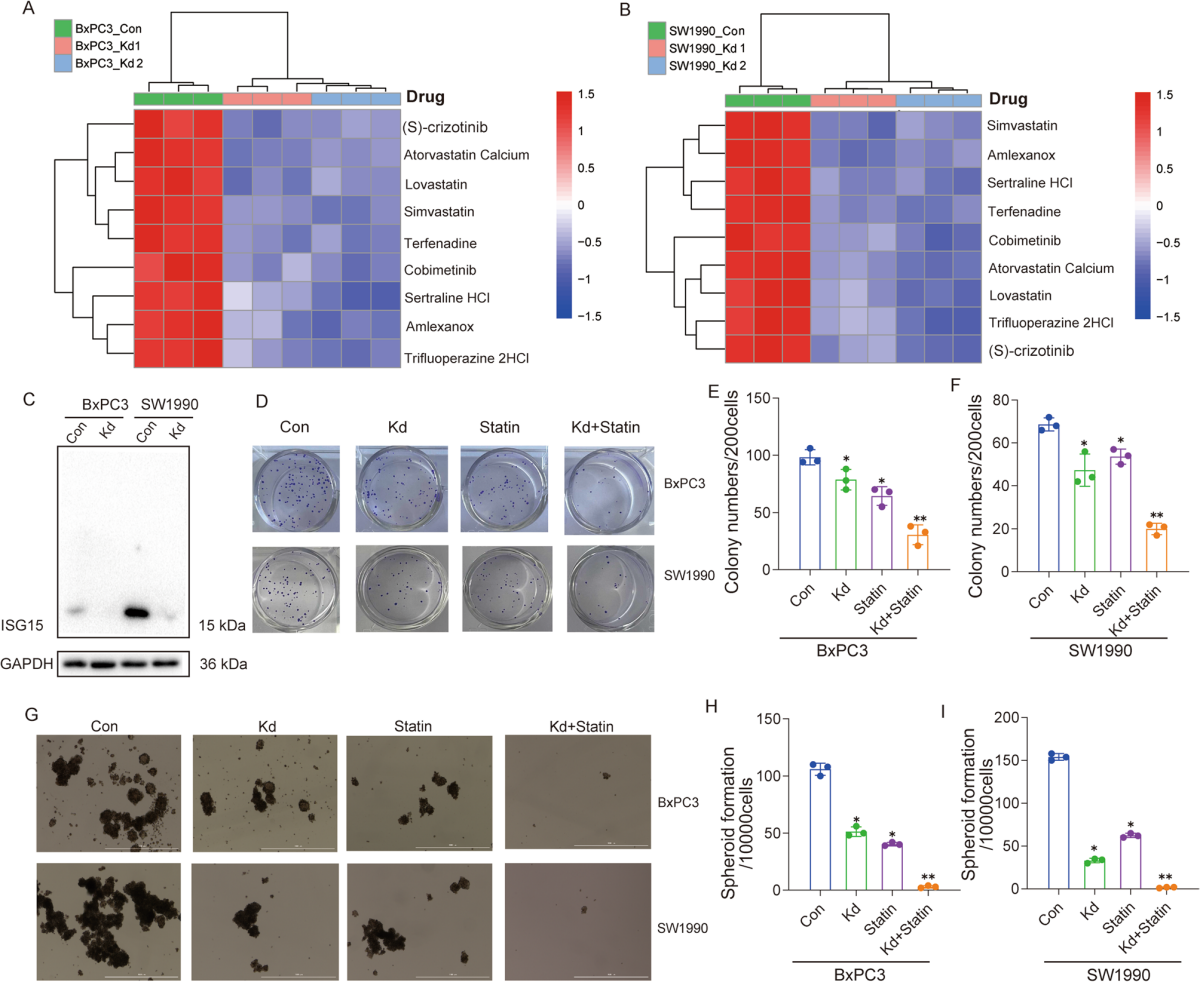
Combined ISG15 Knockdown and Statin Treatment Induces Synthetic Lethality in Pancreatic Cancer Cells. (**A**-**B**) Drug sensitivity analysis of BxPC3 (**A**) and SW1990 (**B**) pancreatic cancer cells treated with drugs, following *ISG15* knockdown (*ISG15 KD*) or control (Con) via CRISPR/Cas9-mediated gene editing. (**C**) Western blot validation of ISG15 protein knockdown efficiency in *ISG15 KD* vs. Con cells. (**D**) Schematic of colony formation assay: 200 single cells/well were treated with statins(20µM), *ISG15* knockdown, or both. (**E**-**F**) Quantification of colony numbers in BxPC3 (**E**) and SW1990 (**F**) cells(*n* = 3 per group, unpaired two-tailed Student’s t test). (**G**) Spheroid formation in serum-free conditions following 10 days of assay. (**H**-**I**) Quantification of spheroid numbers in BxPC3 (**H**) and SW1990 (**I**) cells (*n* = 3 per group, unpaired two-tailed Student’s t test). Data are mean ± SD. *, *P* < 0.05; **, *P* < 0.01; ns, not significant

FDA drug library screening revealed enhanced statin sensitivity in pancreatic cancer (PC) cells upon *ISG15* knockdown (Fig. 1A-B). To validate this association, we generated *ISG15*-knockdown models in BxPC3 and SW1990 cells using lentiviral vectors (Fig. 1C). The CCK-8 assay was conducted to evaluate the combined effects of statins at different concentrations and *ISG15* knockdown (Fig. S2A-D. The results demonstrated that the combination of ISG15 Knockdown and Statin treatment significantly inhibited cell proliferation. Furthermore, flow cytometric analysis of CSC markers CD44 and CD133 indicated that the combined treatment led to a more pronounced reduction in CSC-like features(Fig. S2E).The colony formation assays demonstrated that *ISG15* depletion or statin monotherapy moderately reduced tumor cell survival, whereas their combination synergistically suppressed proliferation (Fig. 1D-F). Strikingly, while *ISG15* silencing or statin treatment alone partially attenuated cancer stem cell (CSC) generation (as assessed by sphere formation), dual targeting nearly abolished spheroid formation capacity (Fig. 1G-I), suggesting synthetic lethality. These results position the *ISG15*-statin axis as a novel therapeutic strategy to eradicate CSC-driven resistance in PC.

### ISG15 knockdown upregulates HMGCR protein expression and confers metabolic vulnerability in pancreatic cancer

**Fig. 2:**
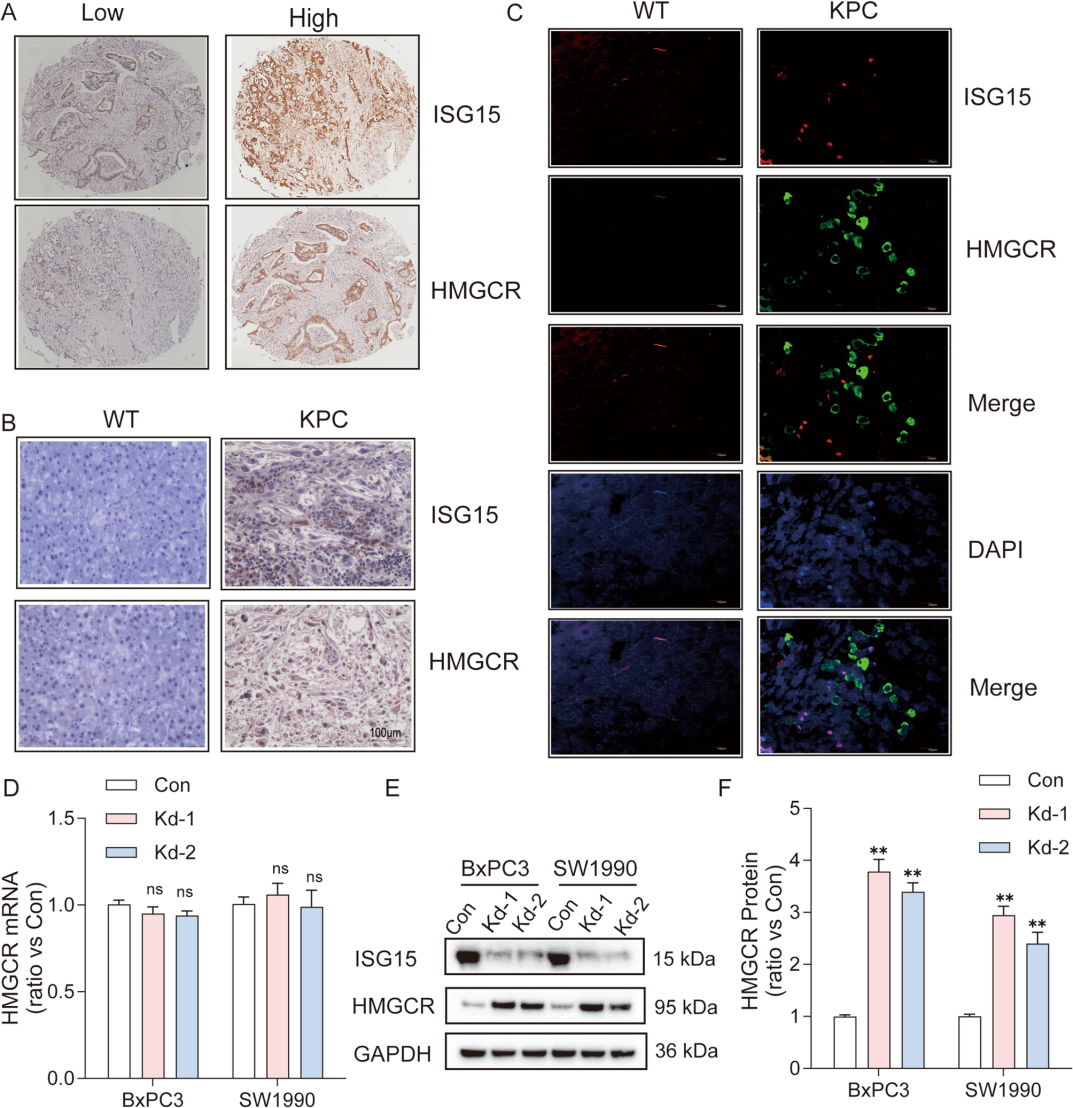
ISG15 Knockdown Stabilizes HMGCR Protein Expression in Pancreatic Cancer. (**A**) Immunohistochemistry (IHC) analysis of ISG15 and HMGCR protein expression in human pancreatic ductal adenocarcinoma (PDAC) tissue microarrays. High ISG15/HMGCR levels correlate with advanced tumor grades. (**B**-**C**) IHC (**B**) and immunofluorescence (IF) staining (**C**) of ISG15 and HMGCR in Kras/p53-driven murine PDAC (KPC) models, confirming co-upregulation but separate expression in malignant tissues. (**D**) qRT-PCR analysis of *HMGCR* mRNA levels in BxPC3 and SW1990 cells with *ISG15* knockdown (KD) vs. control (Con) (*n* = 3 per group, one-way ANOVA followed by Tukey’s test). (**E**-**F**) Western blot (**E**) and quantification (**F**) of HMGCR protein levels in *ISG15* KD cells, demonstrating post-translational stabilization (*n* = 3 per group, one-way ANOVA followed by Tukey’s test). The experiments were performed at least three times. Data are mean ± SD. *, *P* < 0.05; **, *P* < 0.01; ns, not significant

Given the synthetic lethality potential of the ISG15-HMGCR axis, we first assessed their clinical relevance in pancreatic cancer (PC). Tissue microarray analysis revealed that although both ISG15 and HMGCR were highly expressed in pancreatic cancer, their expression displayed a mutually exclusive pattern: tumor cells with elevated ISG15 exhibit low HMGCR levels, and conversely, HMGCR-high cells show minimal ISG15 expression (Fig. 2A). In comparison to wild-type (WT) models, the expression levels of ISG15 and HMGCR were consistently upregulated in the KRAS/p53-driven mouse pancreatic cancer (KPC) models (Fig. 2B). Notably, their expression exhibited distinct spatial heterogeneity, localizing to different ecological niches within the tumor microenvironment, a pattern that mirrors observations in human pancreatic cancer (Fig. 2C). Intriguingly, *ISG15* knockdown (Kd) did not alter *HMGCR* mRNA levels (Fig. 2D) but markedly increased HMGCR protein abundance (Fig. 2E-F), suggesting post-transcriptional regulation.

###  Loss of ISG15 stabilizes HMGCR by blocking ubiquitin-proteasomal degradation in pancreatic cancer independently of its ISGylation

**Fig. 3:**
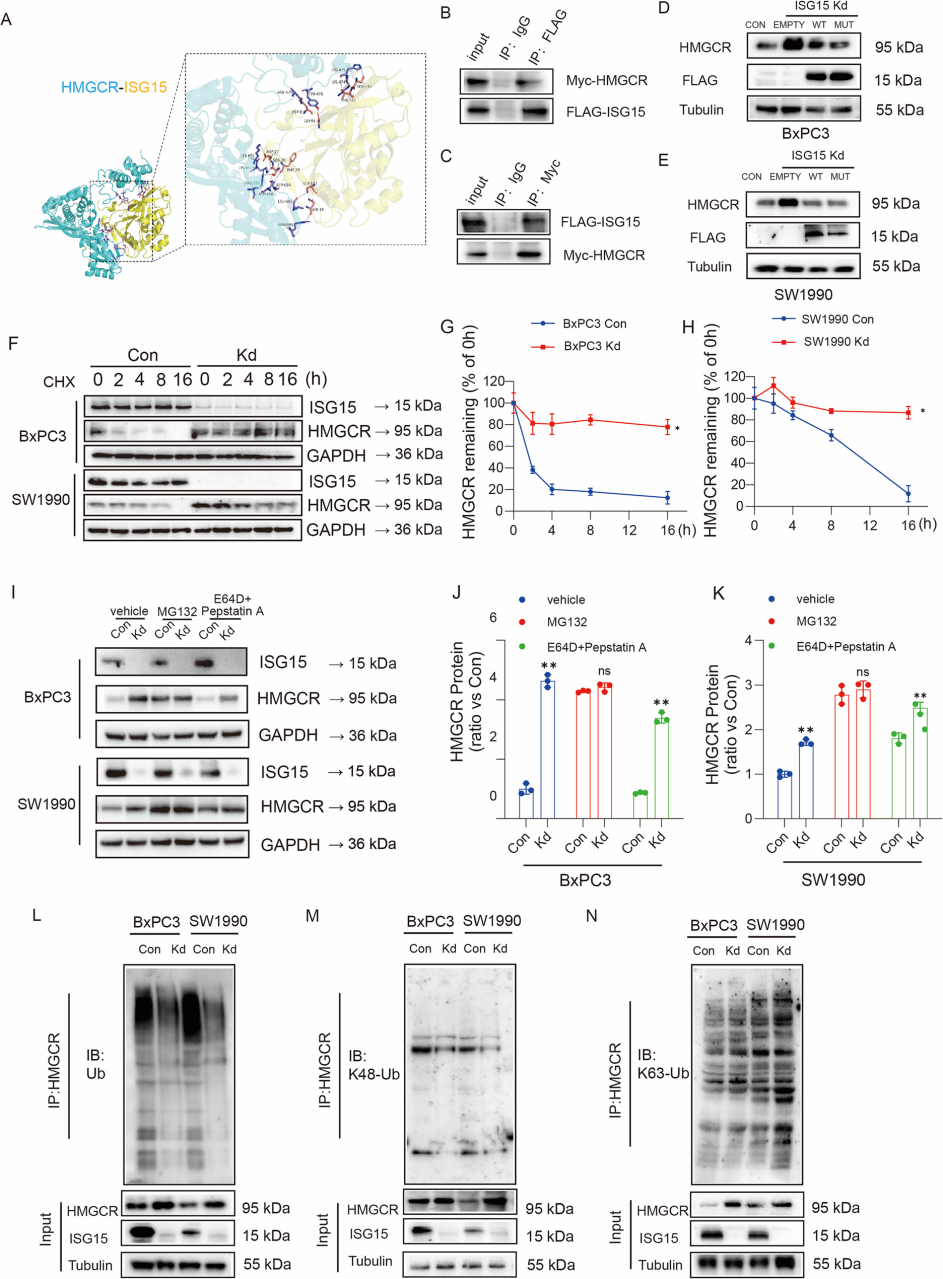
ISG15 Depletion Stabilizes HMGCR Protein by Impeding Ubiquitin-Proteasomal Degradation independently of its ISGylation. (**A**) The binding of ISG15 to HMGCR by molecular docking. (**B**-**C**) CO-IP was used to detect the direct interaction between ISG15 and HMGCR protein. (**D**-**E**) The knocked down ISG15 cells overexpressing ISG15 wild-type and mutant plasmids(G156/157A), and detected the expression of HMGCR protein in Western blot after transfection of BxPC3 and SW1990 cells for 24 h. (**F**) Western blot analysis of HMGCR protein stability in ISG15 knockdown (KD) and control (Con) BxPC3 and SW1990 cells treated with cycloheximide (CHX) to block new protein synthesis. (**G**-**H**) Quantification of HMGCR protein half-life (t₁/₂) in BxPC3 (**G**) and SW1990 (**H**) cells, demonstrating prolonged stability in ISG15 KD cells (*n* = 3 per group, two-way ANOVA with Sidak’s multiple comparisons test). (**I**) Western blot analysis of HMGCR levels in ISG15 KD cells treated with MG132 (proteasome inhibitor) or E64D/Pepstatin A (autophagy-lysosome inhibitor). (**J**-**K**) Quantification of HMGCR protein levels in BxPC3 (**J**) and SW1990 (**K**) cells under proteostatic inhibition. MG132 abolished HMGCR accumulation in ISG15 KD cells, confirming proteasome-dependent degradation (*n* = 3 per group, unpaired two-tailed Student’s t test). (**L**-**N**) Western blot was used to detect the HMGCR of ubiquitination level (**L**), K48 ubiquitin level (**M**) and K63 ubiquitin (**N**) level in ISG15 knockdown BxPC3 and SW1990 cells. The experiments were performed at least three times. Data are mean ± SD, significance determined unpaired Student’s t test or one-way ANOVA followed by Tukey’s test. *, *P* < 0.05; **, *P* < 0.01; ns, not significant

In order to evaluate whether ISG15 has a direct interaction with HMGCR, we predicted the binding of ISG15 to HMGCR by molecular docking, and then used CO-IP to verify the direct interaction between ISG15 and HMGCR (Fig. 3A-C). To determine whether the regulatory effect of ISG15 on HMGCR is dependent on ISGylation, we engineered ISG15 mutants (G156/157A) that lack ISGylation capability. Our findings demonstrated that these ISG15 mutants do not influence the expression of HMGCR, indicating that this regulatory process is independent of ISGylation (Fig. 3D-E). To elucidate the mechanism underlying *ISG15* knockdown-induced HMGCR upregulation, we assessed HMGCR protein stability under proteostatic perturbations. Cycloheximide (CHX)-chase assays revealed that *ISG15* depletion significantly prolonged HMGCR half-life (Fig. 3F-H), indicating enhanced protein stability. Conversely, proteasome inhibition via MG132 abolished the difference in HMGCR levels between control and *ISG15*-knockdown cells (Fig. 3I-K), demonstrating that ISG15 loss specifically impedes HMGCR degradation via the ubiquitin-proteasome pathway. Through further mechanistic investigation, we have demonstrated that the knockdown of ISG15 leads to the degradation of HMGCR via the inhibition of the K48 ubiquitination pathway, while the K63 pathway remains unaffected (Fig. 3L-N).

These findings establish ISG15 as a novel regulator of HMGCR turnover, where *ISG15* silencing disrupts proteasomal targeting, leading to HMGCR accumulation. This stabilization paradoxically sustains cholesterol biosynthesis while depleting metabolic intermediates, creating a therapeutic window for statin-mediated synthetic lethality.

### ISG15 depletion induces cholesterol accumulation and disrupts cholesterol biosynthetic flux in pancreatic cancer

**Fig. 4:**
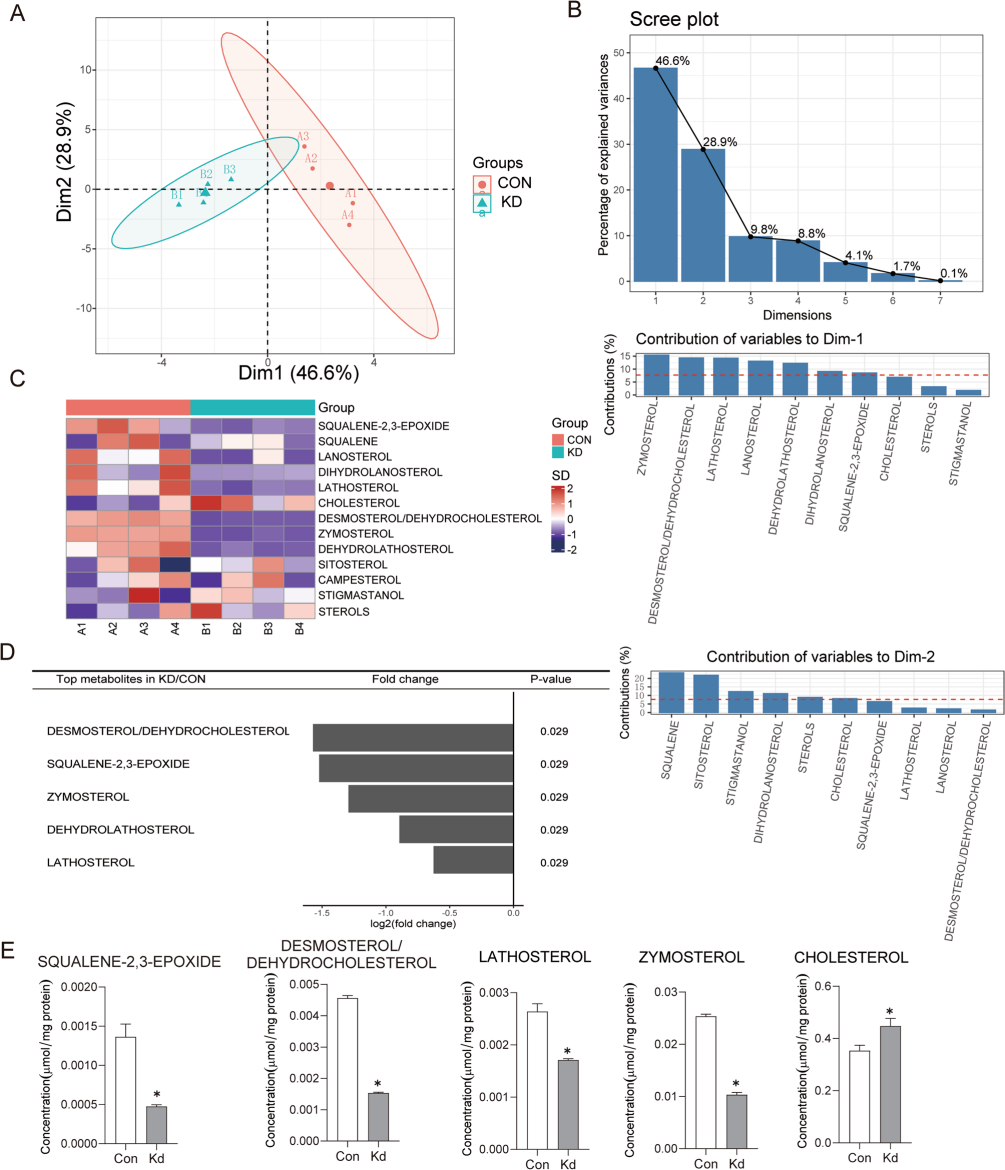
ISG15 Knockdown Rewires Cholesterol Metabolism, Inducing a Metabolic Vulnerability in Pancreatic Cancer. **(A) **Principal component analysis (PCA) of sterolomics profiles in control (Con, Group A) and *ISG15* knockdown (KD, Group B) pancreatic cancer cells. The 95% confidence ellipse confirms distinct metabolic clustering (Dim-1: 46.6%; Dim-2: 28.9%; cumulative variance: 76%) (*n* = 4 per group). **(B)** Loading plot highlighting the top 10 lipids driving PCA separation, dominated by cholesterol biosynthesis intermediates. **(C)** Heatmap of cholesterol-related metabolites, showing global lipid depletion but paradoxical cholesterol accumulation (red) in *ISG15* KD cells (hypothesis test, SQUALENE-2,3-EPOXIDE, *P* = 0.036551699; LANOSTEROL, *P* = 0.042861789; LATHOSTEROL, *P* = 0.024469078; CHOLESTEROL, *P* = 0.034967532; DESMOSTEROL/DEHYDROCHOLESTEROL, *P* = 0.0000495; ZYMOSTEROL, *P* = 0.0000000146; DEHYDROLATHOSTEROL, *P* = 0.008821069). **(D)** Volcano plot identifying differentially abundant metabolites (|log ~ 2 ~ FC| >1, *P* < 0.05) (*n* = 4 per group, Mann-Whitney U test). **(E)** Box plots of the top 4 downregulated cholesterol precursors: desmosterol/dehydrocholesterol, squalene-2,3-epoxide, zymosterol, lathosterol, and also the cholesterol (*n* = 4 per group, unpaired Student’s t test). Each sample was run in 3 technical replicates. Data are mean ± SD. *, *P* < 0.05; **, *P* < 0.01; ns, not significant

To dissect the metabolic consequences of *ISG15* knockdown, we performed sterolomics profiling in pancreatic cancer (PC) cells. Principal component analysis (PCA) revealed clear separation between control and *ISG15*-knockdown groups along the first two principal components (Dim-1: 46.6%; Dim-2: 28.9%), collectively explaining 76% of total variance (Fig. 4A). The 95% confidence ellipse confirmed distinct metabolic clustering without outliers. Loading plots identified the top 10 lipids contributing to Dim-1 and Dim-2, dominated by cholesterol biosynthesis intermediates (Fig. 4B).

Strikingly, heatmap analysis demonstrated that *ISG15* knockdown globally reduced lipid content but paradoxically elevated cholesterol levels (Fig. 4C). Targeted quantification of cholesterol precursors revealed significant downregulation of key intermediates, including desmosterol/dehydrocholesterol, squalene-2,3-epoxide (*p* =, zymosterol, dehydro-lathosterol, and lathosterol (Fig. 4D-E). These data indicate that *ISG15* silencing stalls cholesterol biosynthesis at the HMGCR-dependent step, causing precursor depletion while accumulating end-product cholesterol.

This metabolic rewiring creates a vulnerability: although stabilized HMGCR sustains baseline cholesterol for membrane integrity, the collapse of intermediate flux limits adaptive plasticity, rendering cells dependent on HMGCR activity. This dependency underlies the synthetic lethality observed with statin co-treatment.

### Synthesis and characterization of HA-coated LNP-Kd/Statin nanoplatform

We propose that *ISG15* silencing stabilizes HMGCR protein, likely by impeding ubiquitin-proteasomal degradation, thereby elevating cholesterol biosynthesis to sustain cancer cell survival. However, this compensatory adaptation depletes cholesterol metabolic intermediates (e.g., squalene, zymosterol), rendering cells dependent on HMGCR activity. Consequently, pharmacological HMGCR inhibition (e.g., statins) synergizes with *ISG15* knockdown to exacerbate metabolic stress and eradicate tumors—a vulnerability exploitable for therapy. Synergetic lethality has been observed at the cell level. In order to verify the synergetic lethality in vivo, we used a nano platform for verification.

**Fig. 5:**
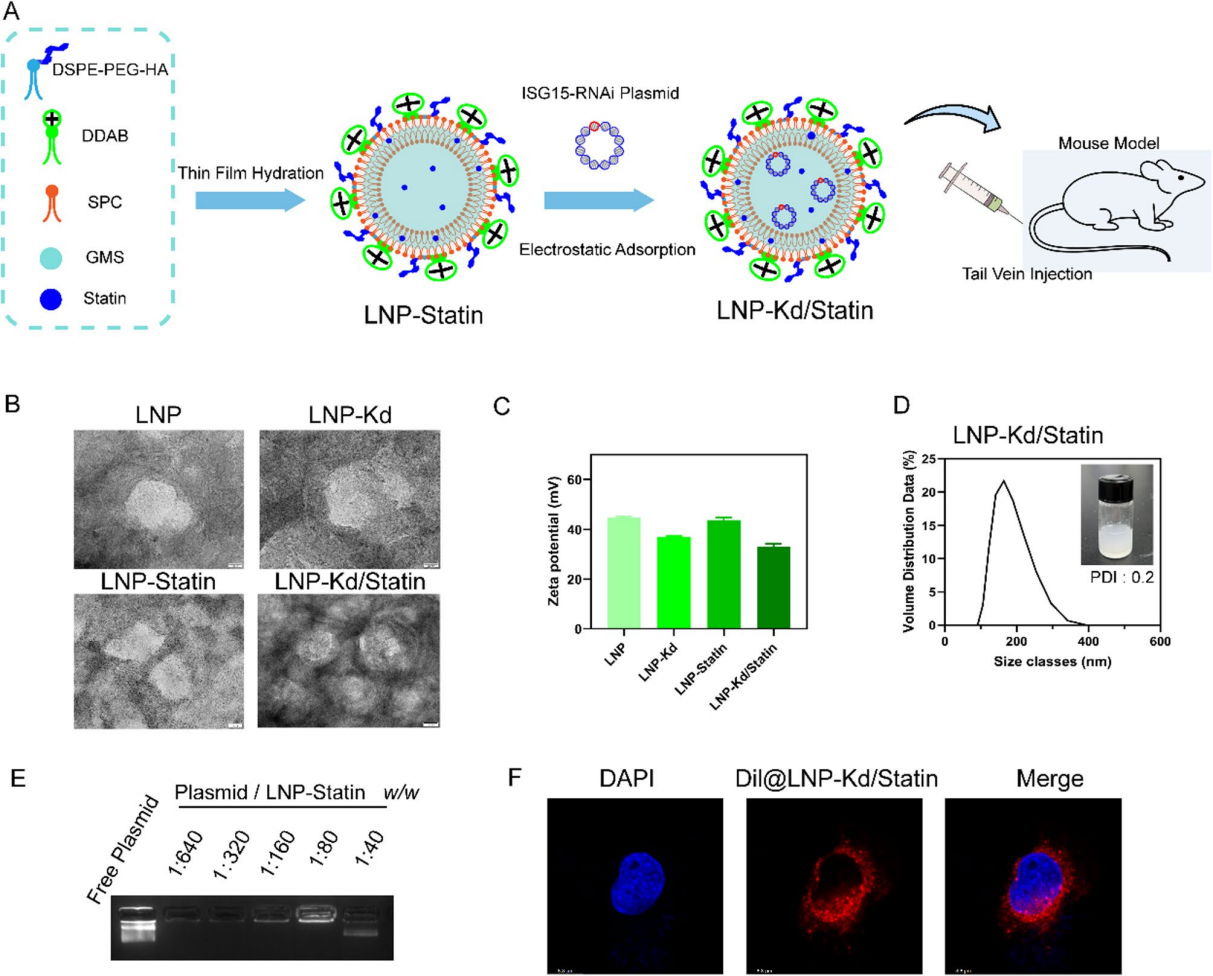
Engineering and Characterization of the HA-Coated LNP-Kd/Statin. **(A)** Nanoplatform Schematic illustration of LNP-Kd/Statin synthesis: Hyaluronic acid (HA)-modified liposomes (LNP-Statin) were functionalized with *ISG15*-targeting siRNA plasmids via electrostatic interactions, yielding the dual-loaded LNP-Kd/Statin. **(B)** Transmission electron microscopy (TEM) images confirming spherical morphology, with a core diameter of 120.7 ± 9.0 nm. Dynamic light scattering (DLS) revealed a hydrodynamic size of 197.7 ± 9.3 nm, reflecting hydration effects. **(C)** Zeta potential analysis: LNP-Kd/Statin exhibited a surface charge of **+ **33.23 ± 0.96 mV, reduced from **+ **43.83 ± 1.01 mV in LNP-Statin due to siRNA neutralization. **(D)** Encapsulation efficiency (EE) and drug loading (DL) for statins (EE: ; DL: ) and siRNA (EE: **~**100%; DL: 1.25%). **(E)** Sustained siRNA release profile over 72 h under physiological conditions. **(F)** Confocal microscopy of Dil-labeled LNP-Kd/Statin (red) internalized by BxPC3 cells (nuclei: blue, DAPI; scale bar: 20 μm). Each sample was run in 3 technical replicates. Data are mean ± SD, significance determined unpaired Student’s t test. *, *P* < 0.05; **, *P* < 0.01; ns, not significant

The hyaluronic acid (HA)-modified liposomal nanoparticle LNP-Kd/Statin was synthesized via a thin-film hydration-sonication method (Fig. 5A). Briefly, statins were encapsulated into HA-functionalized LNPs to form LNP-Statin, which subsequently self-assembled with *ISG15*-targeting siRNA plasmids through electrostatic interactions, yielding LNP-Kd/Statin. Transmission electron microscopy (TEM) confirmed the spherical morphology of LNP-Kd/Statin with an average diameter of 120.7 ± 9.0 nm (Fig. 5B). Dynamic light scattering (DLS) revealed a hydrodynamic size of 197.7 ± 9.3 nm and a zeta potential of **+** 33.23 ± 0.96 mV, compared to **+ **43.83 ± 1.01 mV for LNP-Statin (Fig. 5C). The reduced surface charge of LNP-Kd/Statin was attributed to siRNA plasmid binding, which partially neutralized the cationic lipid DDAB.

Both statins and siRNA exhibited high encapsulation efficiency and drug loading capacity (Fig. 5D-E). To validate cellular uptake, Dil-labeled LNP-Kd/Statin was incubated with BxPC3 cells, and confocal microscopy confirmed efficient intracellular delivery (Fig. 5F). These results demonstrate the successful engineering of a stable, dual-loaded nanoplatform with favorable physicochemical properties for targeted therapy.

### LNP-Kd/Statin nanotherapy suppresses pancreatic cancer organoids and xenografts via synergistic ISG15-HMGCR targeting

To evaluate the therapeutic efficacy of LNP-Kd/Statin, we first validated its biocompatibility: control liposomes exhibited negligible cytotoxicity in BxPC3 cells (Fig. S3), confirming the safety of the delivery system. Subsequent experiments focused on four treatment groups: (1) control, (2) *ISG15* knockdown (Kd), (3) statin monotherapy, and (4) LNP-Kd/Statin combination.

**Fig. 6:**
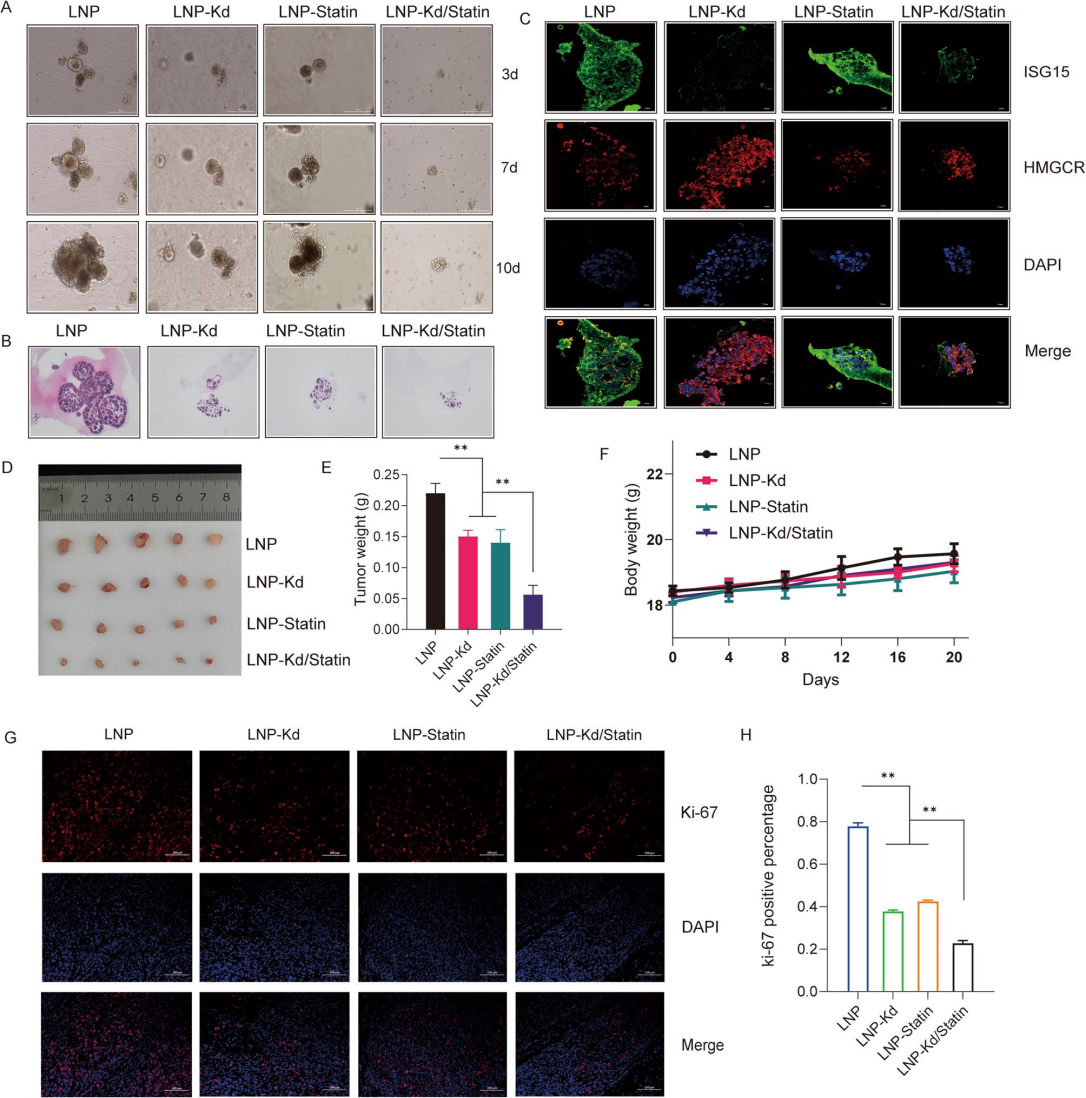
LNP-Kd/Statin Nanotherapy Suppresses Pancreatic Cancer Organoids and Xenografts via Dual Targeting of ISG15 and HMGCR. **(A) **Growth kinetics of BxPC3-derived pancreatic cancer organoids treated with control (Con), *ISG15* knockdown (KD), statin monotherapy (Statin), or LNP-Kd/Statin combination, monitored at days 3, 7, and 10 post-treatment. **(B)** Hematoxylin and eosin (H&E) staining of organoids at day 10, showing disrupted architecture in the combination group (scale bars: 100 μm). **(C)** Immunofluorescence staining confirming ISG15 (green) and HMGCR (red) suppression in LNP-Kd/Statin-treated organoids (nuclei: blue, DAPI; scale bars: 50 μm). **(D) **In vivo therapeutic schema: Nude mice bearing BxPC3 xenografts received tail vein injections of nanotherapeutics starting at day 10 post-implantation. Tumors were harvested at day 28 (*n* = 5 per group). **(E)** Tumor weights across treatment groups (*n* = 5 per group, one-way ANOVA with Tukey’s multiple comparisons test). **(F)** Mouse body weight changes, indicating no systemic toxicity (*n* = 5 per group, one-way ANOVA with Tukey’s multiple comparisons test). **(G)** Immunofluorescence staining of Ki-67 in LNP-Kd/Statin-treated and control groups (*n* = 5 per group, one-way ANOVA with Tukey’s multiple comparisons test). **(H)** Quantification of the positive cell rate of Ki-67 in xenograft tumors (*n* = 5 per group, one-way ANOVA with Tukey’s multiple comparisons test). Data are mean ± SD. *, *P* < 0.05; **, *P* < 0.01; ns, not significant

In BxPC3-derived organoids, *ISG15* knockdown or statin monotherapy moderately reduced growth, while dual treatment induced near-complete suppression (Fig. 6A). Hematoxylin-eosin (HE) staining of organoids at day 7 revealed extensive structural disintegration in the combination group, characterized by loss of epithelial integrity and nuclear fragmentation (Fig. 6B). Immunofluorescence further confirmed that LNP-Kd/Statin simultaneously silenced ISG15 protein and inhibited HMGCR expression (Fig. 6C), underscoring target engagement.

In vivo, subcutaneous BxPC3 xenografts in nude mice showed that LNP-Kd/Statin combination therapy significantly reduced tumor volume **(**65% reduction vs. control, *p* < 0.01) and weight (58% decrease, *p* < 0.01), outperforming monotherapies (Fig. 6D-E). Notably, mouse body weights remained stable (Fig. 6F), indicating minimal systemic toxicity. Post-treatment tumor analysis confirmed coordinated downregulation of ISG15 and HMGCR proteins (Fig. S4), aligning with in vitro findings. The Ki67 test of tumors confirmed that the combination group had a better effect on the treatment of tumors (Fig. 6G-H), and our formulation had no organ toxicity (Fig. S5 and Table S1).

These results demonstrate that LNP-Kd/Statin achieves synthetic lethality by dual targeting of ISG15 and HMGCR, effectively eradicating CSCs and bulk tumor cells while preserving systemic tolerability.

## Discussion and conclusion

### Synthetic lethality targeting cholesterol regulation via ISG15 and HMGCR

Pancreatic ductal adenocarcinoma (PDAC), ranking as the third leading cause of cancer-related deaths [27], remains a therapeutic enigma. Despite incremental advances in chemotherapy and immunotherapy [28–32], the resilience of cancer stem cells (CSCs) and metabolic adaptability of PDAC cells continue to drive therapeutic failure. Here, we unveil a dual-pronged strategy that merges synthetic lethality with nanotechnology to dismantle CSC-driven resistance and metabolic plasticity, a paradigm shift in the management of PDAC.

Unlike prior studies focusing on ISG15’s roles in autophagy or immune evasion [14, 33, 34], we identify its novel function as a regulator of cholesterol flux. By coupling *ISG15* knockdown with HMGCR inhibition, we exploit a synthetic lethal interaction that collapses cholesterol biosynthesis intermediates (e.g., squalene oxide, zymosterol) while paradoxically elevating cholesterol, a metabolic “double bind” that starves CSCs of plasticity-enhancing precursors yet saturates membranes to limit survival signaling. This mechanism transcends traditional single-target approaches, uniquely leveraging metabolic stress to eradicate CSC reservoirs.

### A targeted nanoplatform for dual delivery and stromal penetration

While liposomal irinotecan and nab-paclitaxel have validated nanomedicine in PDACs [23–25], our HA-coated LNP-Kd/Statin platform represents a quantum leap. By co-delivering *ISG15*-siRNA and statins, we achieve tumor-specific targeting via CD44 receptor-mediated uptake [35–37], bypassing the dense stromal barrier that plagues conventional therapies [38, 39]. The system’s capacity to achieve dual targeting of ISG15 (via RNAi-mediated silencing) and HMGCR (through statin inhibition) while circumventing systemic toxicity addresses a pivotal unmet need in pancreatic ductal adenocarcinoma (PDAC) therapy: enabling multi-target precision without the cumulative off-target effects characteristic of conventional combinatorial regimens.

### Immunometabolic crosstalk and spatial tumor heterogeneity

Notably, despite the concurrent upregulation of ISG15 and HMGCR in pancreatic cancer, their expression is spatially segregated, with ISG15-high and HMGCR-high tumor cells occupying distinct niches in a mutually antagonistic manner. Our findings extend beyond metabolic rewiring. ISG15 knockdown reduced PD-L1 expression and enhanced CD8⁺ T-cell infiltration [33], suggesting immune sensitization, a feature absent in current statin therapies. This reciprocal expression suggests potential compensatory pathways or functional antagonism between ISG15-mediated immune regulation and HMGCR-driven metabolic reprogramming in pancreatic tumorigenesis. This dual immunometabolic action positions LNP-Kd/Statin as a bridge between targeted therapy and immunotherapy, potentially overcoming the immunosuppressive PDAC microenvironment [28–32].

### Toward clinical translation: combination strategies and biomarker-driven therapy

The synthetic lethality of ISG15-HMGCR inhibition offers a blueprint for mechanism-guided combination therapy. Unlike traditional high-throughput drug screens, our approach rationally pairs a ubiquitin-proteasome modulator (ISG15) with a metabolic enzyme inhibitor (statin), maximizing tumor-specificity. Building on these findings, subsequent research should prioritize three strategic avenues to accelerate clinical impact. First, biomarker-driven trials are essential to correlate baseline ISG15/HMGCR expression with therapeutic response, enabling patient stratification for precision intervention. Second, stromal reprogramming strategies, such as combining LNP-Kd/Statin with hyaluronidase to degrade hyaluronic acid-rich extracellular matrix, could enhance nanodrug penetration and target engagement in desmoplastic tumors. Third, leveraging ISG15’s dual role in PD-L1 suppression and CD8⁺ T-cell recruitment opens avenues for immunotherapy synergy, where LNP-Kd/Statin may prime immunologically "cold" PDAC microenvironments for checkpoint inhibitor efficacy. Together, these approaches could dismantle the multifaceted barriers to PDAC treatment, transforming synthetic lethality from concept to clinic.

In conclusion, by integrating synthetic lethality, nanotechnology, and sterolomics, we pioneer a metabolic-targeted strategy that cripples PDAC’s Achilles "heel"—its dependence on cholesterol plasticity. This work not only redefines ISG15 as a linchpin of PDAC metabolism but also establishes a blueprint for next-generation nanotherapies designed to outmaneuver therapeutic resistance. As precision oncology pivots toward multi-targeted, microenvironment-aware strategies, our platform exemplifies how mechanistic depth and engineering innovation can converge to transform outcomes in intractable malignancies.

## Supplementary Information

Supplementary Material 1.

## Data Availability

The authors confirm that the data supporting the findings of this study are available within this main text and the supplementary materials. Raw data are stored in the laboratory of the corresponding authors and are available upon request.
