# Supplementary material for: The simultaneous targeted Inhibition of ISG15 and HMGCR disrupts cancer stemness through metabolic collapse and induces synthetic lethality in pancreatic ductal adenocarcinoma
Source: J Exp Clin Cancer Res. 2025 Dec 9;44:317. doi: 10.1186/s13046-025-03561-x (PMC12690959; doi:10.1186/s13046-025-03561-x)
Supplement: Supplementary file 1 — Supplementary Material 1. [file 13046_2025_3561_MOESM1_ESM.docx]

**Supporting Information**

**Figure S1. Standard Curve Validation for Statin Quantification**
Linear regression analysis of UV-vis absorbance (290 nm) versus statin concentration (7.81–500.00 μM) demonstrates a robust correlation (R² = 0.998), validating the precision of drug quantification in the LNP-Kd/Statin formulation. The equation y = 0.012x + 0.045 defines the calibration curve, where y represents absorbance and x denotes statin concentration.

**Figure S2.** **Combined ISG15 Knockdown and Statin Treatment inhibits proliferation and stemness in pancreatic cancer cells**

**(A-B) BxPC3 (A) and SW1990 (B) pancreatic cancer cells were treated with statins for 36 hours(n = 3 per group，unpaired two-tailed Student’s t test). Cell proliferation and cytotoxicity were analyzed using CCK-8 absorbance. (C-D) Statin sensitivity in BxPC3** **(C) and SW1990 (D) cells following CRISPR/Cas9-mediated ISG15 knockdown (ISG15 KD) or control treatment (Con), with statin exposure for 36 hours. Cell viability and cytotoxicity were evaluated using CCK-8 assay (n = 3 per group，unpaired two-tailed Student’s t test). (E) Flow cytometric analysis of CSC markers (CD44 and CD133) in BxPC3 and SW1990 cells to evaluate tumor stemness index.**

**Figure S3. Biocompatibility of Control Liposomes in Pancreatic Cancer Organoids**
**(A)** Schematic of cytotoxicity assay: BxPC3-derived organoids were treated with blank liposomes (control) or LNP-Kd/Statin for 36 hours. CCK-8 viability analysis revealed no significant cytotoxicity in the control liposome group (98.3 ± 3.2% viability vs. untreated, ns), confirming the biocompatibility of the delivery vehicle. Data are mean ± SD(n = 3 per group, one-way ANOVA followed by Tukey’s test). Data are mean ± SD. *, *P<0.05*; **, *P*<0.01; ns, not significant.

**Figure S4. LNP-Kd/Statin Suppresses ISG15 and HMGCR in Pancreatic Tumors**
**(A)** Immunofluorescence was used to detect the expression of ISG15 (red) and HMGCR (green) in xenograft tumors from mice treated with LNP-Kd/Statin or control. **(B)** Western blot analysis of ISG15 and HMGCR protein levels in xenograft tumors from mice treated with LNP-Kd/Statin or control. **(C)** Quantification of ISG15 and HMGCR protein levels in the same xenograft tumors as in (B).Dual targeting reduced ISG15 by **72%** (P < 0.01) and HMGCR by **65%** (P < 0.01), confirming in vivo mechanism engagement (n = 3 per group，unpaired two-tailed Student’s t test). Data are mean ± SD.*, *P < 0.05*; **, *P < 0.01*; ns, not significant.

**Figure S5. LNP-Kd/Statin is non-organotoxic to mice**

**(A)**  HE staining of heart, liver, spleen, lung and kidney treated with LNP-Kd/Statin or control.
